# Transcriptomic responses to prion disease in rats

**DOI:** 10.1186/s12864-015-1884-7

**Published:** 2015-09-05

**Authors:** Allen Herbst, Anthony Ness, Chad J. Johnson, Debbie McKenzie, Judd M. Aiken

**Affiliations:** Department of Agricultural, Food and Nutritional Science, Centre for Prions and Protein Folding Diseases, University of Alberta, Edmonton, AB T6G 2M6 Canada; Department of Biological Sciences, Centre for Prions and Protein Folding Diseases, University of Alberta, Edmonton, AB T6G 2M6 Canada; Department of Medicine, School of Medicine and Public Health, University of Wisconsin, Madison, WI 53706 USA

## Abstract

**Background:**

Prions diseases are fatal neurodegenerative diseases of mammals. While the molecular responses to prion infection have been extensively characterized in the laboratory mouse, little is known in other rodents. To explore these responses and make comparisons, we generated a prion disease in the laboratory rat by successive passage beginning with mouse RML prions.

**Results:**

We describe the accumulation of rat prions, associated pathology and the transcriptional impact throughout the disease course. Comparative transcriptional profiling between laboratory mice and rats suggests that similar molecular and cellular processes are unfolding in response to prion infection. At the level of individual transcripts, however, variability exists between mice and rats and many genes deregulated by prion infection in mice are not affected in rats.

**Conclusion:**

Our findings detail the molecular responses to prion disease in the rat and highlight the usefulness of comparative approaches to understanding neurodegeneration and prion diseases in particular.

**Electronic supplementary material:**

The online version of this article (doi:10.1186/s12864-015-1884-7) contains supplementary material, which is available to authorized users.

## Background

Prion diseases are an unusual class of fatal, transmissible, neurodegenerative disorders that affect the mammalian central nervous system. They are caused by the accumulation of an abnormal conformation of the normal host encoded cellular prion protein, PrP^C^. Although the exact mechanism is unknown, this conformational rearrangement of PrP^C^ is thought to be brought about by template-directed misfolding wherein seed molecules of the abnormal isoform, PrP^Sc^, convert PrP^C^ into new PrP^Sc^ molecules. Laboratory investigation of prion diseases typically relies upon rodents, which can be infected with natural isolates of scrapie [1] albeit with some difficulty as ovid scrapie isolates need time to adapt to rodents. This adaptation is characteristic of interspecies transmission of prion infections and reflects the molecular adaptation that must occur to allow interaction between exogenous foreign PrP^Sc^ and host PrP^C^ molecules, selecting for conformations which exhibit efficient template-directed prion-specific folding. In some cases, no conformational solution is found, reflecting a molecular species barrier to disease transmission.

In recent years, advances in genomic technologies have allowed unprecedented examination of the transcriptional responses induced by prion infection. These studies [2–9] have the aim of identifying pathways underlying the mechanism of prion-induced neurotoxicity. A second important aim has been to identify signature molecules that might act as surrogate biomarkers for these diseases as there are significant analytical challenges associated with sensitively detecting and specifically distinguishing PrP^Sc^ from normal PrP^C^. This work has most commonly been performed using laboratory mice as cross-sectional time course experiments can be performed, including the important pre-clinical asymptomatic phase of disease. Critically, however, the relevance and generalizability of mouse prion responses to prion diseases of other species is unknown. To begin addressing these issues, we adapted mouse RML prions into rats with the aim of identifying common prion disease transcriptional responses. Brain tissue from control and scrapie affected rats was transcriptionally profiled and genes whose expression was changed in response to prion diseases identified. We identified 712 genes differentially regulated in rat prion disease. Only 33 % of the rat genes with changes in expression were found to have similar responses in the corresponding mouse orthologs, questioning the universality of previous mouse gene expression profiles. This comparative approach allowed the identification of genes whose prion-induced expression change is conserved between mice and rats and highlights the significance of these genes. We show that Rat-Adapted Scrapie (RAS) is a powerful tool for an -omics based approach to decipher the molecular impact of prion disease *in vivo*, with applicability to the molecular mechanisms of disease and biomarker discovery.

## Results

### Development of rat-adapted scrapie

**Fig. 1:**
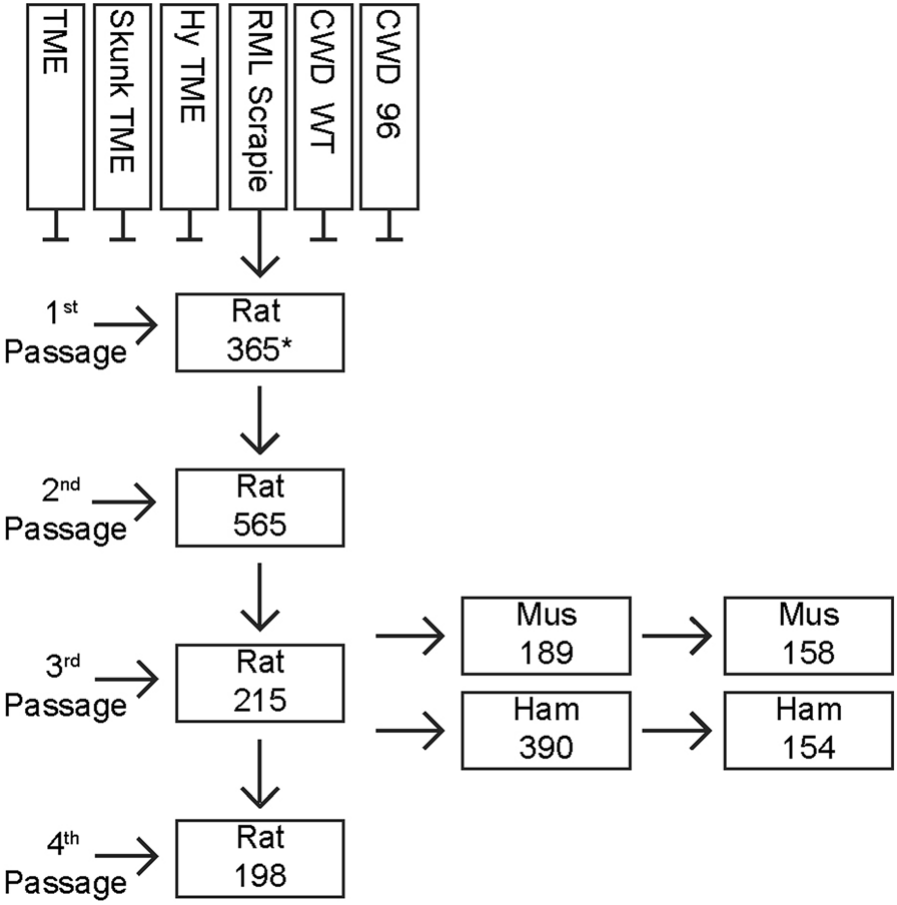
Interspecies transmission of prion disease. Six prion agents were used for initial transmission to rats including transmissible mink encephalopathy (TME), skunk-adapted TME, hamster-adapted TME strain Hyper, Rocky Mountain Laboratories (RML) mouse-adapted scrapie, chronic wasting disease (CWD) from wild-type deer, and CWD from wt/96S deer. After 1 year of incubation, first passage rats were euthanized to determine the extent of PrP-res accumulation. PrP-res was observed solely in rats infected with RML scrapie prions. This agent was serially passaged until becoming rat-adapted, as indicated by the decrease in and stabilization of the incubation period. Third passage rat-adapted scrapie transmitted to mice and hamsters with 100 % penetrance

To develop a rat-adapted strain of prion disease, we introduced 6 different prion disease agents, Chandler/RML from mice, Stetsonville transmissible mink encephalopathy (TME), Hyper from hamsters, skunk-adapted TME and Chronic Wasting Disease (CWD) from wild type and 96S deer [1, 10–13] into rats (Fig. 1). Of these primary transmissions, only RML mouse scrapie induced the accumulation of proteinase K resistant PrP (PrP-res) after 1 year of incubation as determined by western analysis (10 % brain homogenates and phosphotungstic acid-enriched brain homogenates). Second passage of rat-adapted RML resulted in clinically affected rats at 565 days post-inoculation (dpi). Upon subsequent serial passage, the incubation period stabilized at ~200 days. Clinical symptoms of prion disease in the rat consisted of ataxia, lethargy, wasting, kyphosis, and myoclonus. Prion-affected rats also show porphyrin staining around their head.

**Fig. 2:**
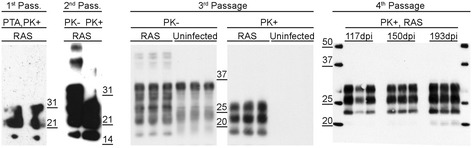
Accumulation of PrP-res in rat-adapted scrapie (RAS). Brain homogenates from each passage were assayed for the presence of protease resistant PrPPrP-res by immunoblot. PrP-res was observed following phosphotungstic acid (PTA) enrichment at first passage. Di- and mono- glycosylated bands were the most abundant isoforms of PrP-res. Uninfected age-matched controls are shown for 3^rd^ and 4^th^ passage animals. In fourth passage, PrP-res accumulated to substantial levels by 117 days post-infection (dpi)

**Fig. 3:**
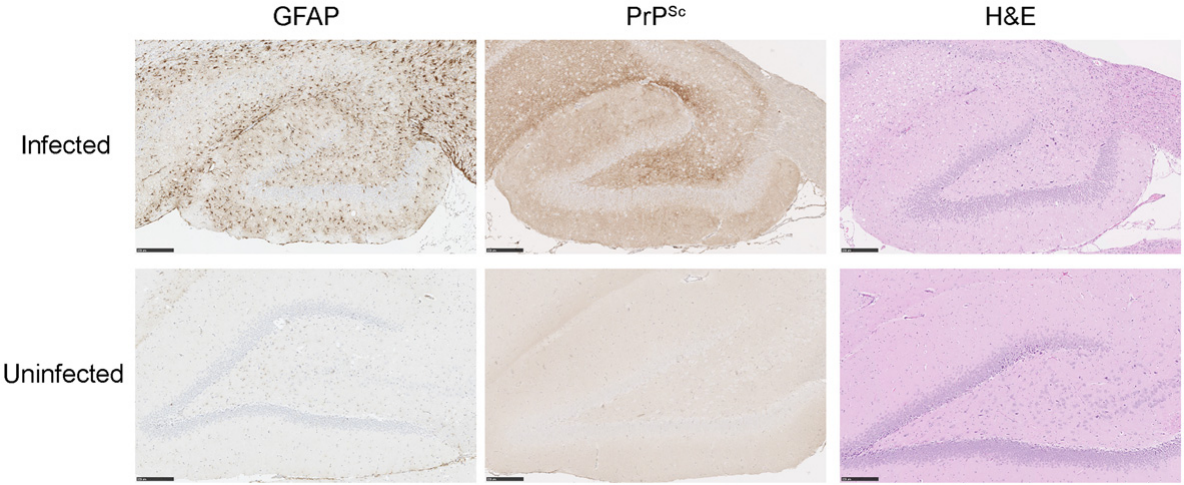
Histological analysis of rat-adapted scrapie in the hippocampus at clinical disease. Infected animals showed intense immuno-staining for deposits of PrP-res and GFAP expressing astrocytes. Spongiform change is an abundant feature in rat prion disease. The scale bar represents 250um

PrP-res was detected in all infected animals by both immunoblot (Fig. 2) and immunohistochemistry (Fig. 3). Di- and mono-glycosylated bands were the most abundant isoforms of PrP^Sc^ (Fig. 2). PrP^Sc^ was extensively deposited in the cerebral cortex, hippocampus, thalamus, inferior colliculus and granular layer of the cerebellum (Additional file 1: Figure S1). GFAP-expressing activated astrocytes were found throughout the brain, particularly in the white matter of the hippocampus, thalamus and cerebellum (Additional file 1: Figure S2). Spongiform lesions were a predominant feature of clinical rat disease (Fig. 3).

### Gene expression profiling of rat-adapted scrapie

**Fig. 4:**
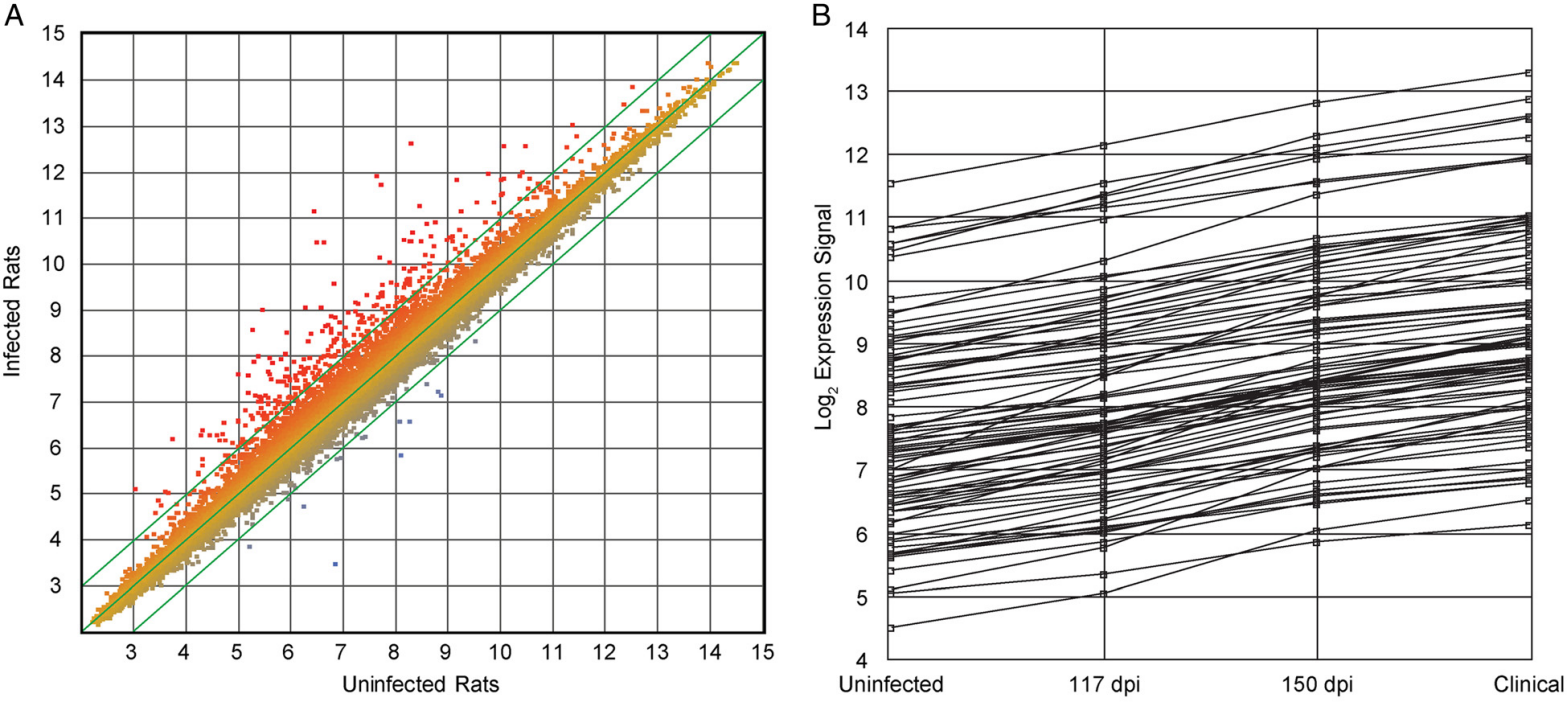
Gene expression profile from rats clinically affected with prion disease. **a**. The expression levels of individual genes from uninfected and rats clinically affected with prion disease were plotted. The axis is the expression level from uninfected rats and the y-axis is the corresponding expression level of the gene in infected rats. The green lines indicate 2-fold changes in gene expression. Any points outside of these lines are genes whose expression is deregulated greater than 2-fold. **b**. Cluster of 81 genes whose expression is up-regulated throughout disease. Clustering was performed using 291 genes whose expression was changed more than 2-fold and with a statistical confidence interval of 90 % using a false positive rate approach. Ontological analysis of this gene cluster is consistent with the involvement of these genes in neuroinflammation. Gene expression profiles were obtained from infected and control rats at three time points. Comparisons between uninfected rats at the three time points did not identify statistically significant changes in gene expression

To define the molecular pathology of rat-adapted scrapie, gene expression profiling was performed on brain tissue from infected and age-matched control rats at three time points. Rat cohorts were sacrificed at two preclinical time points, 113 and 150 days post-inoculation, as well as during clinical disease (198 days). At the clinical disease, 712 genes were differentially regulated within a 95 % confidence interval (Additional file 2: Table S1) and 367 genes were differentially expressed by greater than 2-fold (Fig. 4). Significant genes and their orthologs were used for pathway analysis using DAVID Bioinformatics Resource [14] (Additional file 3: Table S2, Additional file 1: Figure S3). Pathway analysis suggested that the gene expression profile was consistent with immune activation and maturation as well as inflammation (Additional file 2: Table S1), an interpretation supported by the observable GFAP positive astrocytes (Fig. 3) and gliosis associated with prion disease. Other pathways highlighted by the analysis include an increase in the transcripts of lysosomal and endosomal components. Many genes changes that were observed at clinical time points were also up-regulated earlier in disease, during the preclinical stage (Fig. 4b). Validation of gene expression was performed using TaqMan qPCR assays (Additional file 1: Figure S4). Data are deposited in the NCBI GEO repository under the accession number GSE63930.

### Comparative transcriptomics

**Fig. 5:**
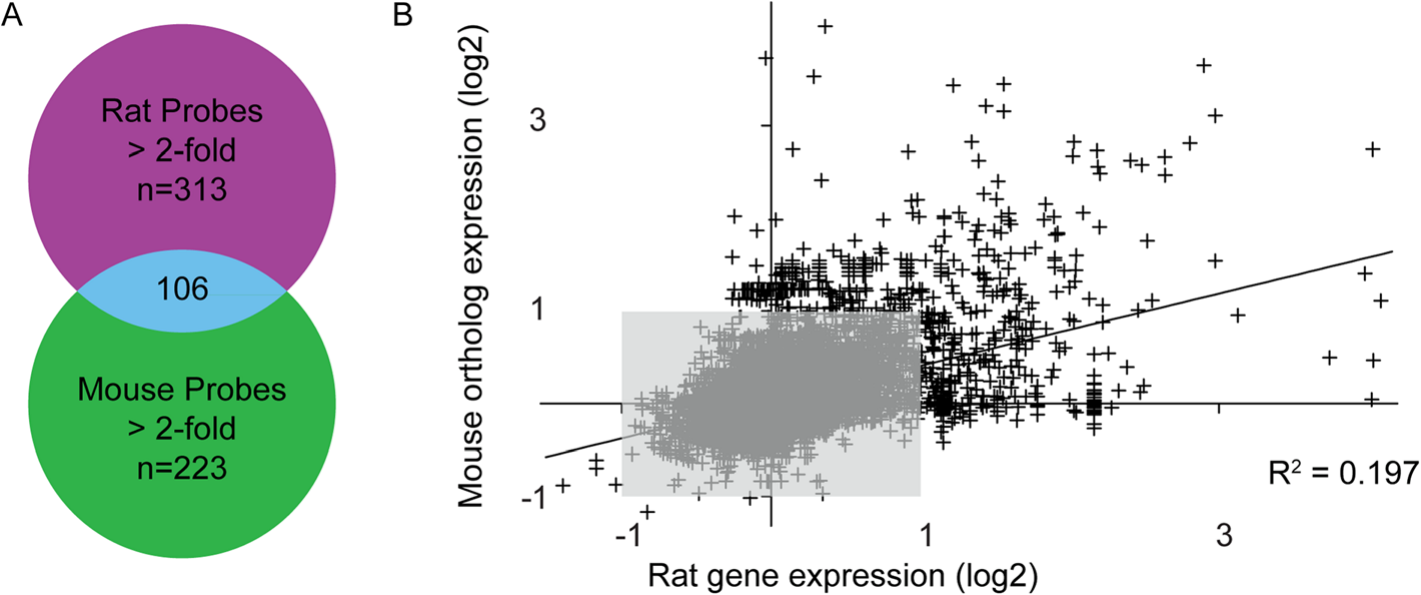
Comparison of genes upregulated in rat and mouse prion disease. **a**. Orthologous probe sets differentially regulated by 2-fold were identified in both rat and mouse prion disease and the overlap determined. Duplicate probe sets measuring the same gene were eliminated for simplicity. **b**. Mouse scrapie induced gene expression changes were regressed onto changes induced by rat scrapie. Expression values are log2 transformed. The grey box indicates those orthologs whose expression is not changed >2-fold. Expression of probe sets that lie along the axes are highly divergent in prion disease between mice and rats. Stricter bioinformatic mapping of orthologous pairs did not meaningfully improve the regression

To further explore the gene expression data, differentially expressed probe sets in rat adapted scrapie were compared with their mouse orthologs in RML scrapie and vice versa. Raw mouse gene expression data was downloaded from prion.systemsbiology.net [6], normalized and analyzed similarly to the rat data. Mouse RML prion responses at clinical (22 and 23 weeks post- infection) and two preclinical time points (16 and 18 or 10 and 12 weeks post-infection) were selected to correspond, fractionally, to our rat time points, 113, 150 and 198 dpi. A high degree of correlation between expression fold changes was not observed (Fig. 5). To examine comparative gene expression responses qualitatively, three approaches were used. In one strict analysis, gene expression responses from orthologs with “best match” probe sets (3700 orthologous pairs) were compared. Of these 3700 probe sets, 22 were deregulated greater than 2-fold in rat scrapie at clinical disease. Of these 22, half were also deregulated at least 2-fold in mouse scrapie. In a broader analysis, 32,625 orthologous rat-mouse probe sets were identified using Ensembl BioMart [15, 16]. Of these mouse-rat probe set pairs, 250 were deregulated greater than 2-fold in mouse scrapie at clinical disease. 149 of the 250 were deregulated greater than 2-fold in rats. In a final analysis, 333 genes whose expression is deregulated in mouse prion disease were examined for their correlation to rat disease. This 333 gene set is representative of the mouse prion disease transcriptome and was derived from experiments profiling prion disease responses in multiple backgrounds of mice, including transgenically modified mice and multiple strains of mouse scrapie prions [6]. Assigning and subsequent mapping of the 333 mouse genes to rats using Ensembl BioMart results in 499 probe set pairs, reflecting 288 rat genes. Of the 499 mouse probe sets whose expression is changed in response to mouse scrapie, only 317, 234 and 202 rat probe sets are changed greater than 1.5-, 1.8- and 2-fold respectively. We conclude that many genes deregulated in rat scrapie are not differentially expressed in mouse scrapie and vice versa. For example, cathepsin E is up-regulated 5.6-fold in rat scrapie, but not differentially expressed (1.1-fold) in mouse scrapie. Similarly, scrapie responsive gene 1 [17, 18] was up-regulated 1.6-fold in mouse scrapie, but not differentially expressed (1.1-fold) in rat scrapie. Additional file 4: Table S3 includes a refined list of prion disease responsive transcripts conserved in rodent scrapie. Despite these differences at the level of some individual genes, the overall gene expression profiles were similar. Mapping deregulated genes from both mice and rats to gene ontology terms identified the same biological processes. Visualization of these gene ontology terms and their order of significance using tree mapping [19] (Additional file 1: Figure S3) demonstrates the overall similarity between gene expression profiles between mice and rats.

## Discussion

Mice have been the preferred laboratory rodent for prion diseases research because they can be inexpensively housed and are amenable to transgenesis, facilitating short incubation periods upon over expression of PrP or examination of non-murine prion replication by exogenous PrP expression. Subsequent to the sequencing and annotation of the mouse genome and the development of high density transcriptional arrays for measurements of gene expression profiling, mice have been used extensively to examine the molecular pathology of prion disease probing the impact of prion and animal strain. To expand upon this foundation, we adapted mouse prions to rats. We reasoned that by taking a comparative approach to the molecular pathology of prion disease, inferences into the variability of the molecular response to prion diseases could be obtained. Finally, rats, like mice, can be used in a time course study of prion disease. This allows for the identification of early transcriptional responses to prion infection, responses which are particularly valuable for the identification of surrogate disease biomarkers.

To generate rat prion disease, we attempted to adapt six different prion disease agents to rats (Fig. 1). Of these six agents, only mouse RML prions were able to surmount the molecular species barrier to prion disease transmission. Mouse PrP differs from the rat by ten amino acid changes, seven in the mature protein and four in the N-terminal octa-peptide repeat region (Additional file 1: Figure S5.) As rat shares the most prion protein sequence identity with the mouse, is perhaps not surprising that the mouse-adapted agent transmitted whereas the other prion agents did not. The lack of transmission of the other agents may be explained based upon divergent amino acid residues that inhibit conversion of rat PrP^C^. For example, at position 113, mice and rats share a common leucine whereas the other prions tested contain methionine.

Our adaptation of mouse scrapie into rats appears similar to that process observed by Kimberlin and Walker [20]. The increase in incubation period at second passage is due to our sacrificing first passage rats at 365 days post-inoculation despite a lack of clinical symptoms, whereas Kimberlin and Walker allowed the first passage animals to reach clinical disease. Slight increases in reported incubation periods in our experiments compared to the Kimberlin transmission, are due to different criteria for euthanizing animals; Kimberlin and Walker sacrificed rodents at the onset of clinical disease. The similarity of our rat-adapted scrapie with the Kimberlin rat transmission is supported by back passage and interspecies transmission experiments. Back passage of RAS to mice reestablished a strain that was indistinguishable from RML/Chandler with an incubation period of 158 days. Transmission of RAS into hamsters resulted in an incubation period of 154 days post infection after second passage. The passage of rat agent into mice and hamsters bears resemblance to that of Kimberlin and Walker and supports the conclusion that the two rat prion infections are similar. By third passage, the rat prions were fully adapted. Subsequent passages did not result in significant incubation period shortening and gene expression data from third passage clincially affected rats is substantially identical to fourth passage rats.

Rat-adapted scrapie exhibits features common to other prion diseases. A long preclinical phase precedes a rapid clinical period marked by a progressive neurological dysfunction with prominent ataxia, kyphosis and wasting. The protease resistant PrP observed is typical as is the widespread deposition of PrP^Sc^ in the brain. Spongiosis and reactive astrogliosis are as expected of a prion disease. Gene expression profiles from rats clinically affected with prion disease revealed a strong neuronal inflammation associated with proliferated and activated astrocytes. This is perhaps best observed through the up-regulation of GFAP, a hallmark of the molecular response to prion infection. GFAP was up-regulated 1.7, 2.6 and 3.5 fold at the 2 preclinical and clinical time points in rats.

Strategies comparing gene expression between mice and rats were undertaken to explore the level of conservation of prion disease transcriptomic responses. Analysis of both narrow and broad subsets of genes were undertaken to remove selection bias. Gene subsets were selected based upon stringent similarities in oligonucleotide probe design including identical gene regions in rat and mouse and high percent identity between orthologous probe pairs. In many cases, probes were identical. Broader examination of gene subsets were also undertaken to ascertain, more globally, the degree of similarity between the transcript profiles. Finally, mouse genes whose transcriptional responses were deregulated across different prion strain and mouse background combinations and considered central to prion disease were examined. Our comparative analyses of gene expression changes between mouse RML and rat-adapted scrapie suggest that there are differences in gene expression in response to prion disease at the level of individual transcripts, despite the fact that the overall response is neuro-inflammatory and many processes are conserved. Immune response processes are the most significant gene ontology categories identified in both mice and rats. This is consistent with the observed neuroinflammation that accompanies prion disease progression and pathogenesis.

One interpretation of the observed differences between mice and rats could be based upon strain phenomena. Prion strains result from conformational differences in the tertiary structures of PrP^Sc^. Different strains of prions have different biochemical and biological properties, thus they might also elicit different molecular responses, observable in transcriptome type experiments. Gene Expression profiles of 301 V and RML on the same mouse background, however, show similar transcriptomic responses to prion disease [6]. The variations observed in response to different prion strains are smaller than those observed between different strains of mice which are smaller than those observed in different species. Similarly recent comparisons of 22 L and RML mouse prion infections, which affect different brain regions, identified strikingly similar gene expression responses [9]. Further, it is unlikely that evolution would have selected for distinct molecular neurodegenerative responses to different prion strains. At the whole brain level, species specific differences seem more important.

## Conclusions

In this study, we describe the transmission and adaptation of mouse scrapie prions into rats. Comparative analysis of gene expression changes in mice and rat prion disease identified differences in gene expression of the level of individual genes, despite the fact that the overall profile is neuro-inflammatory and conserved. These data suggest caution in extrapolating solely from mouse prion gene expression data to prion infections in other species especially at the level of individual transcripts. Our data further show the utility of the laboratory rat as a useful prion disease research tool and demonstrate the power of comparative transcriptional profiling to explore the systems biology of prion diseases.

## Methods

### Ethics statement

This study was carried out in accordance with the recommendations in the NIH Guide for Care and Use of Laboratory Animals and the guidelines of the Canadian Council on Animal Care. The protocols used were approved by the Institutional Animal Care and Use Committees at the University of Wisconsin and University of Alberta.

### Rat transmission and adaptation

Prion agents used were the Rocky Mountain Lab (RML) strain of mouse-adapted scrapie, Stetsonville Transmissible Mink Encephalopathy [10] (TME), Hyper (Hy) strain of Hamster TME [11], 1st passage Skunk-adapted TME [12] prepared by transmission of TME into skunks and CWD from genetically defined deer transmissions [13]. Six rats were infected with each prion at first and second passage.

Brains from animals clinically affected with prion disease were aseptically removed and prepared as 10 % (w/v) homogenates in sterile water. 10 % brain homogenate (50uL) was inoculated intracranially into weanling Wistar rats. After 1 year of incubation, preclinical rats from all infections were euthanized by CO2 inhalation and the brain excised, homogenized and analyzed by western blot. PrP^Sc^ positive samples (from the mouse RML transmission) were re-inoculated into naive animals. Subsequent serial passages were from rats clinically affected with rat-adapted scrapie.

Brains from rat passages were aseptically removed and bisected sagitally. Brain halves were reserved for immunohistochemistry (IHC) in formalin, or frozen on dry ice for immunoblotting, RNA isolation or subsequent passage. For IHC, tissues were fixed in 10 % buffered formalin, followed by antigen retrieval at 121C, 210 KPa in 10 mM citrate buffer for 2 min. After cooling to room temperature, sections were treated with 88 % formic acid for 30 min and 4 M guanidine thiocyanate for 2 h. Endogenous peroxidases were inactivated by 0.03 % hydrogen peroxide and tissue was blocked with 1 % normal mouse serum. Mouse anti-PrP mAB SAF83 (Cayman Chemical) was biotinylated and applied at a 1:250 dilution in blocking buffer overnight at 4C. Washes were performed in 10 mM PBS with 0.05 % Tween-20. Streptavidin-peroxidase (Invitrogen) was added for DAB color reaction. Anti-GFAP immunohistochemistry was performed as above but with the formic acid and guanidine treatment steps excluded. Mouse anti-GFAP mab G-A-5 (Sigma), was used at a 1:400 dilution.

Brain samples for immunoblot were treated with or without Proteinase K (Roche) at a ratio of 3.5ug PK/100ug protein at 50ug/mL for 30 min at 37C. Phosphotungstenic acid enrichments were performed as described [21, 22]. Bis-Tris SDS-PAGE was performed on 12 % polyacrylamide gels (Invitrogen) and transferred to PVDF. Immunoblotting was performed using mABs SAF83 (Cayman Chemical), 6H4 (Prionics), or 3 F10 (a kind gift from Yong-Sun Kim), all at a 1:20,000 dilution.

### Gene expression profiling

RNA was extracted from frozen brain halves from infected and control animals with the QIAshredder and RNeasy mini kit (Qiagen, Valencia, CA) in accordance with the manufacturer’s recommendations. Due to the relatively large mass of rat brain, an initial homogenization was performed with a needle and syringe in 5 mL of buffer RLT, before further diluting an aliquot as per the manufacturer’s instructions. RNA was examined using a bioanalyzer(Agilent Technologies, Santa Clara, CA) to ensure the quality of isolated RNA. Total RNA was amplified and labeled in preparation for chemical fragmentation and hybridization with the MessageAmp Premier RNA amplification and labeling kit (Life Technologies, Grand Island, NY). Amplified and labeled cRNAs were hybridized on Affymetrix (Santa Clara, CA) rat genome 230 2.0 high density oligonucleotide arrays in accordance with the manufacturer’s recommendations. Three biological replicates were examined from infected and control rats at clinical disease following third passage and from each time point (113, 150 and 198 days post infection) of the fourth passage. Quantitative PCR validation assays were performed on cDNA samples by probe cleavage assays. RNA samples were normalized to the relative amounts of GAPDH and β-Actin. Three biological replicates and three technical replicates were analyzed for each gene and at each time point.

### Data analysis

Differentially Expressed Genes were identified using Arraystar 5.0 (DNA Star, Madison, WI). Robust multiarray normalization using the quantile approach was used to normalize all microarray data. A moderated *T*-test with a multiple comparison adjustment [23] was used to reduce the false discovery rate, yet preserve a meaningful number of genes for pathway analysis. Pathway analysis was performed using the DAVID Bioinformatics database [14]. Comparative analysis of genes induced by prions in mouse [6] and rat disease was performed on probe set matches generated by the Affymetrix comparison spreadsheets and matches obtained by using ENSEMBLE biomart release 78 [15, 24].

### Availability of supporting data

The data sets supporting the results in this article are available in the NCBI GEO repository under accession GSE63930.

## Additional files

Additional file 1: Figures S1-S5. (PDF 1365 kb) Additional file 2: Table S1. Genes whose expression is differentially regulated in rat-adapted scrapie. Genes were selected for inclusion if their dysregulation was greater than 1.6-fold at clinical disease and their F-test p-value was less than 0.05 using a Benjamini-Hochberg false discovery rate correction for multiple testing. (XLSX 117 kb) Additional file 3: Table S2. Ontology terms associated with genes differentially expressed in rat-adapted scrapie. (XLSX 97 kb) Additional file 4: Table S3. Prion disease responsive probe sets conserved between mice and rats. Genes with significant differential expression (*p* < 0.05) and fold changes greater than 1.8 were selected from mice and rats. (XLSX 47 kb)
