# Supplementary material for: Transcriptomic responses to prion disease in rats
Source: BMC Genomics. 2015 Sep 5;16(1):682. doi: 10.1186/s12864-015-1884-7 (PMC4560926; doi:10.1186/s12864-015-1884-7)
Supplement: Additional file 1: Figures S1-S5. — (PDF 1365 kb) [file 12864_2015_1884_MOESM1_ESM.pdf]

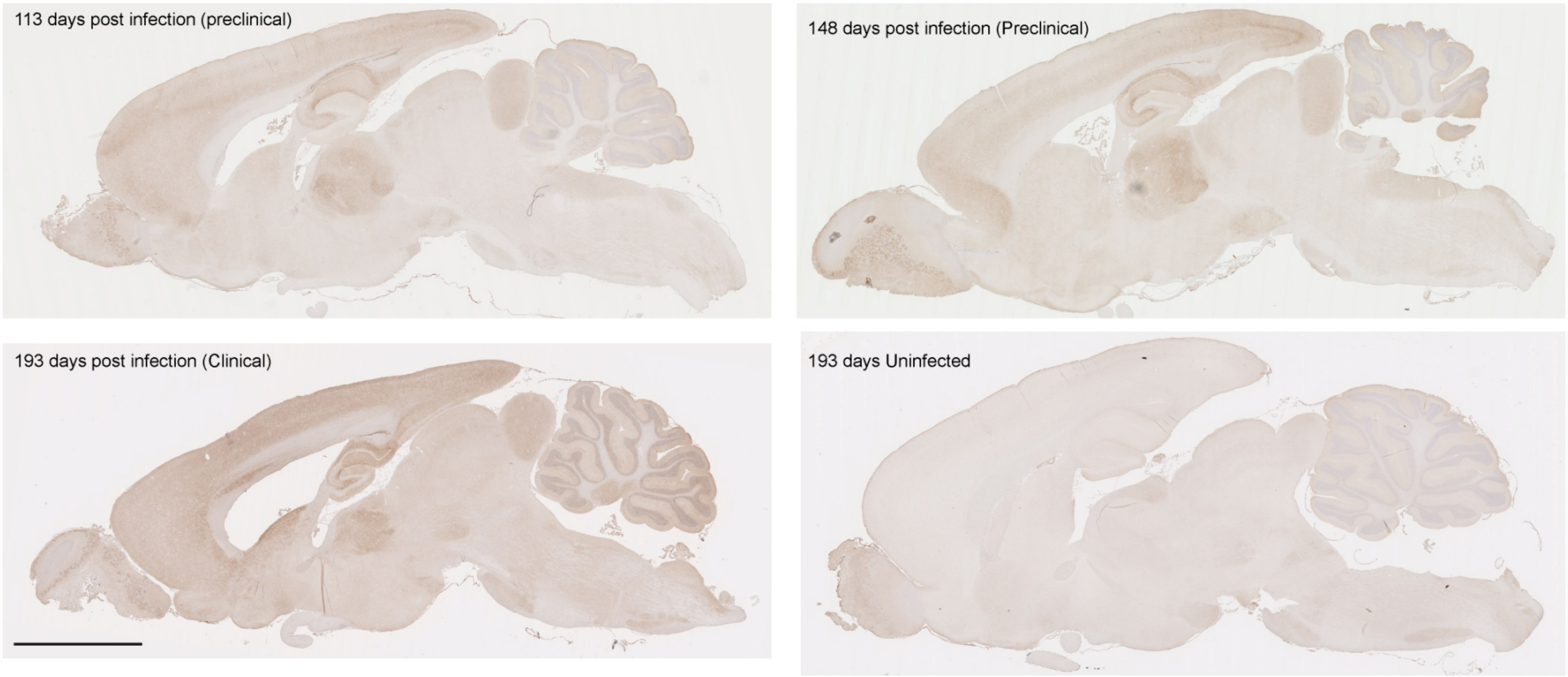

Additional Figure 1. Accumulation and distribution of PrP-res in the rat brain during prion disease. Immunohistochemical staining for PrP-res on sagital sections. The scale bar in the lower left is 5mm.

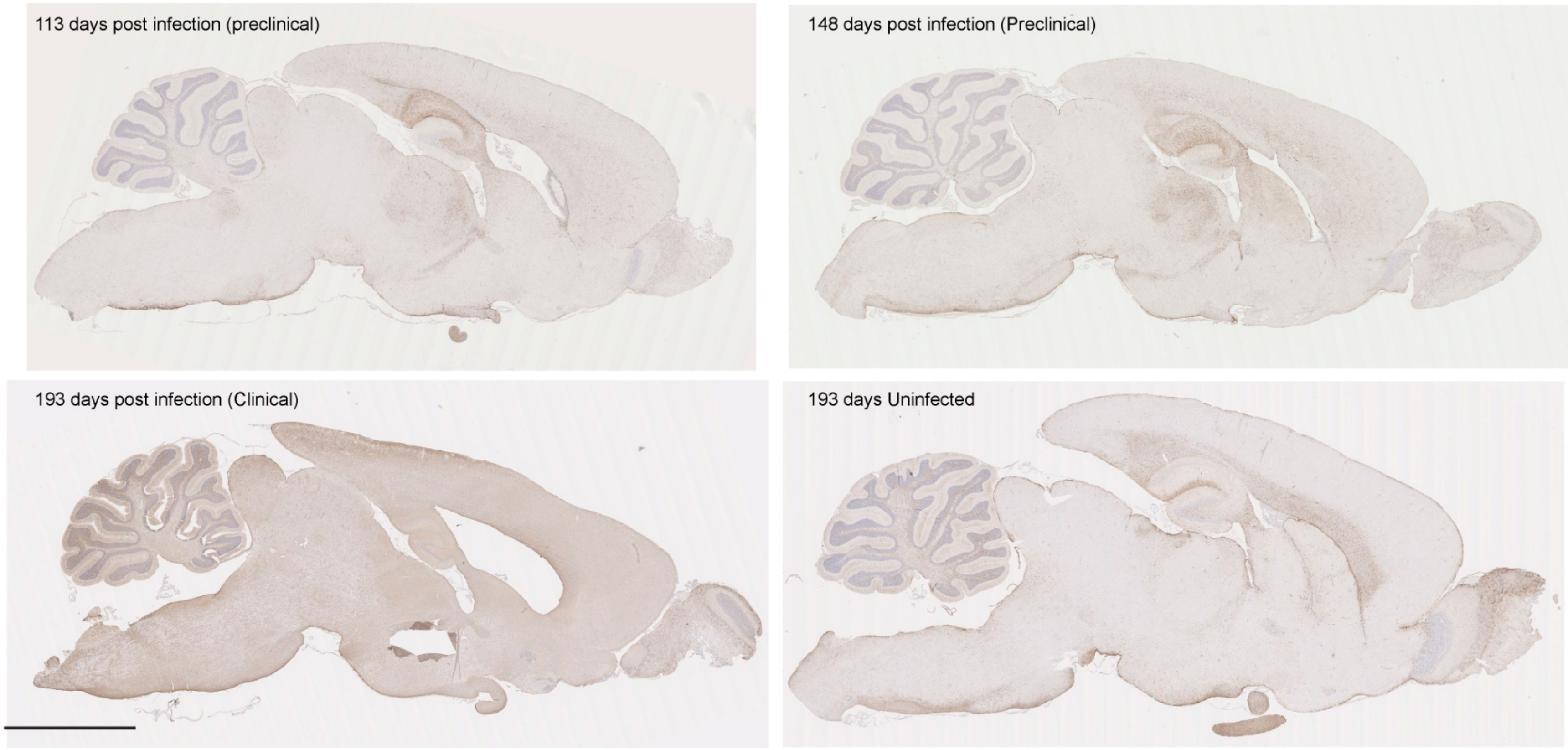

Additional Figure 2. Accumulation and distribution of GFAP in the rat brain during prion disease. Immunohistochemical staining for GFAP on sagital sections. The bar in the lower left is 5mm.

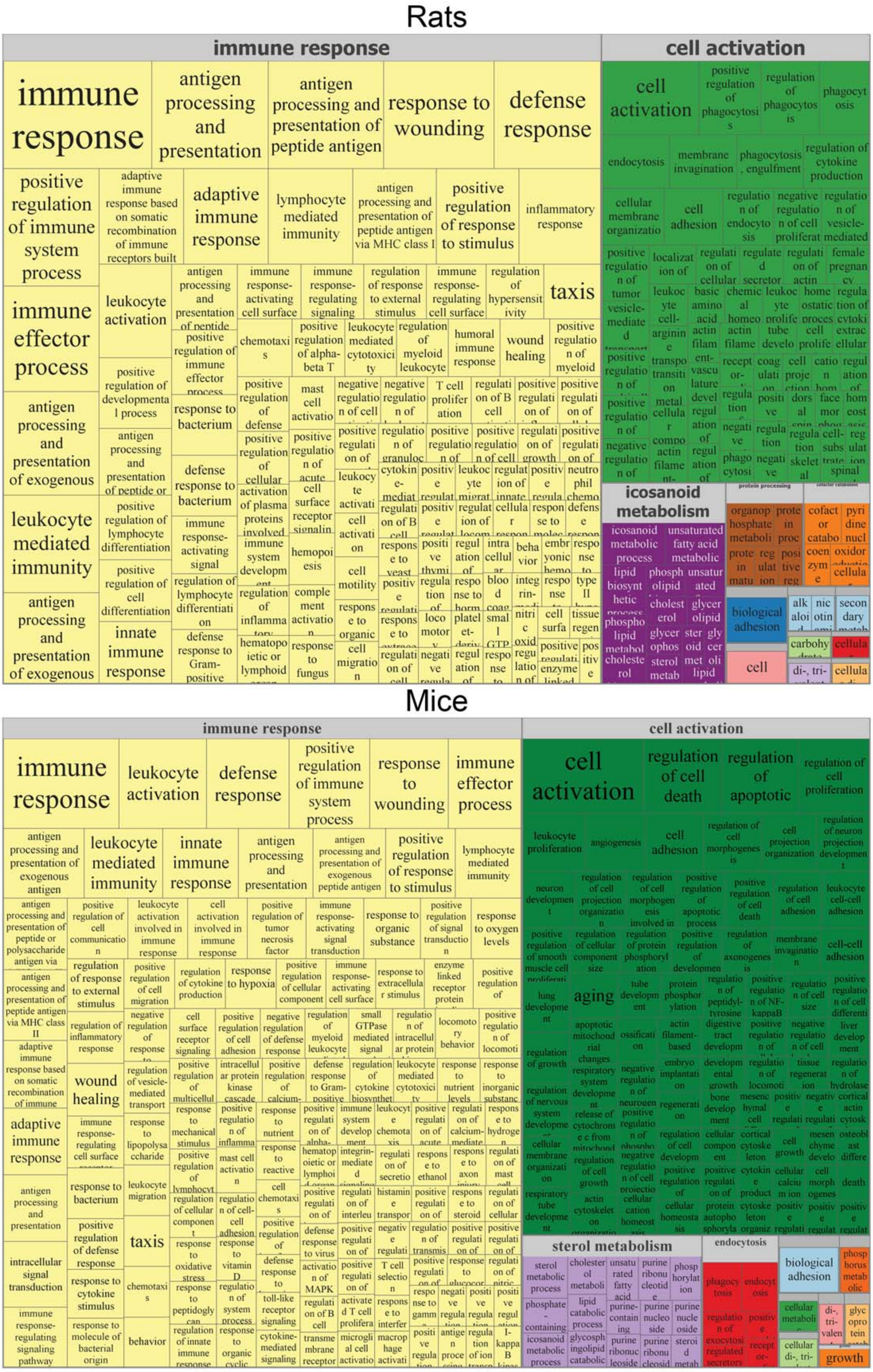

Additional Figure 3. Genes expressed in clinically affected prion infected mice and rats map to similar gene ontology. A Tree mapping approach was used to visualize gene ontology terms associated with prion disease in mice and rats. The area of each rectangle corresponds to the level of significance of enrichment of each GO term. The largest response to prion infection in both mice and rats is activation of immune-related processes. Whether the similar profile is a result of cross-species bioinformatic assignment of gene ontology terms based upon homology or generic nonspecific ontological terms remains unknown. The convergence of pathways and processes between rat and mice prion diseases likely reflects the changes in cell population as indicated by the proliferation and activation of astrocytes and microglia.

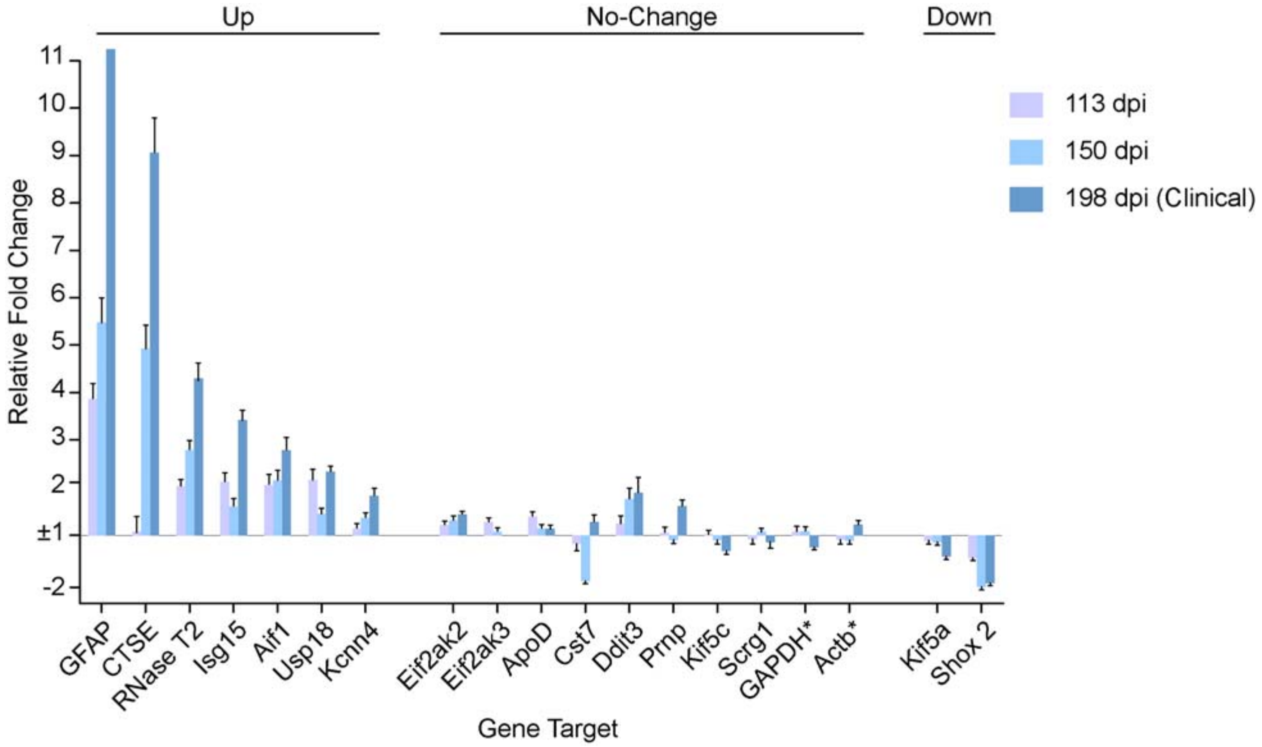

Additional Figure 4. Gene expression changes were validated in 19 genes by quantitative PCR. Genes whose expression was expected to be up or down regulated and unchanged were selected for validation. Gene expression changes were normalized using the expression of both β-actin and GAPDH except where the expression of β-actin or GAPDH were measured*, in which case the controls were normalized using each other. Gene expression was validated using quantitative PCR at two preclinical time points, 113 and 150 days post infection and at clinical disease 198days post infection.

A.

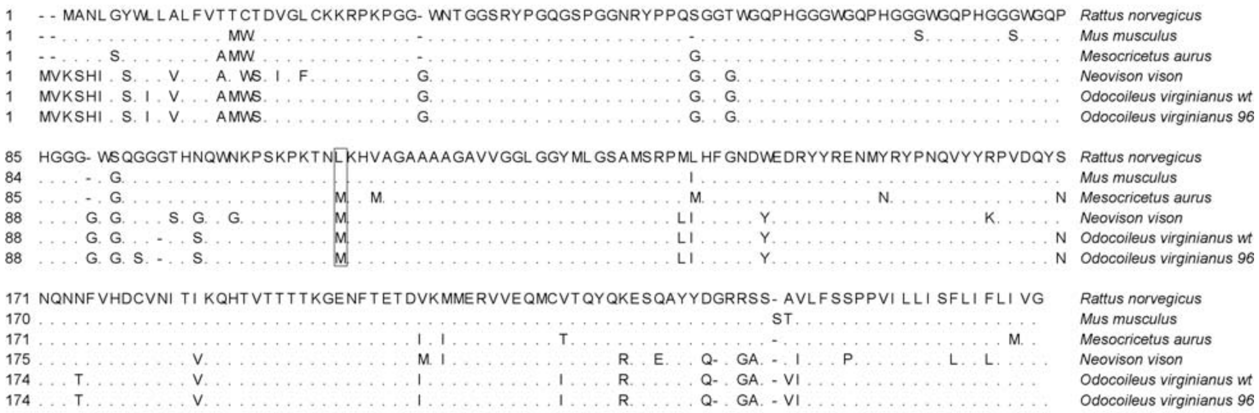

B.

Percent Identity (upper) / Divergence (lower)

	1	2	3	4	5	6		
**1**		96.9	94.1	86.2	86.6	86.2	**1**	*Rattus norvegicus*
**2**	2.8		94.1	85.8	88.2	87.8	**2**	*Mus musculus*
**3**	6.2	5.8		85.8	87.4	87.0	**3**	*Mesocricetus aurus*
**4**	14.8	14.9	15.3		93.0	92.2	**4**	*Neovison vison*
**5**	13.0	11.6	11.6	7.4		99.6	**5**	*Odocoileus virginianus wt*
**6**	13.5	12.1	12.1	7.8	0.4		**6**	*Odocoileus virginianus 96*
	**1**	**2**	**3**	**4**	**5**	**6**		

Additional Figure 5. A. Sequence alignment of rat prion protein with mouse, hamster, mink, and white-tail deer. The boxed residues at amino acid 113 shows the sole difference between mouse prions that successfully adapted to rats and those agents which did not transmit. B. Percent homology between rats and sources of prion used.
